# Bibliographical Notices

**Published:** 1854-12

**Authors:** 


### BIBLIOGRAPHICAL NOTICES.

*Notes of M. Bernard's Lectures on the Blood; with an Appendix.* By WALTER F. ATLEE, M. D. Philadelphia: Lippincott, Grambo & Co. 1854.

This work constitutes "The publication of Notes taken at the Lectures of M. Claude Bernard on the Blood, delivered at the College of France in the winter of 1853–54. To which, as an appendix, are added some notes derived from the Lectures of Mr. Charles Robin, and from practical study under him.

We confess that our expectations were much raised, when the work with the above title and announcement was placed in our hands.

The experiments and labors of M. Bernard were well known; and his contributions to Physiological Science had enriched the teachings and lectures of our Schools.

It was, however, only by his occasional communications in the Comptes Rendus, in the proceedings of the Biological Society, and by a few monographs, as well as by the reports of those who had employed the opportunity of attending his lectures and witnessing his experiments, that the medical profession in this community had been made acquainted with his views. The late contributions by Donaldson in the American Journal of the Medical Sciences, had increased our familiarity with his researches in regard to the Pancreas, Liver, and some other points of interest. To combine all these views and investigations, and present them in a condensed form, was a desideratum which it was hoped the work before us would supply.

The distinguished lecturer to whom it is dedicated, in his letter quoted in the Preface, would seem to have entertained some such expectation, when, having adverted to the prominence of the subjects and the eminence of the authorities, he writes, "You will be able to lay before us the whole of this subject, posted up to the last moment."

And the author himself informs his readers, "This little work contains the *last word*, of French Science at least, in regard to the Anatomy and Physiology of the circulatory system."

M. Bernard proposes to study the Blood, a subject, as he states, of incontestible importance under a point of view *particularly physiological*, by which he appears to mean the phenomena presented in the circulation by experiments on living animals, and we are, therefore, not to expect much to be contributed to the Chemical History of that fluid.

Great attention is shown to its physical characters, color, &c. It is insisted, that the difference in the color of arterial and venous blood is not a vital distinction, as the bright red hue may be produced by the administration of the oxide of carbon, a most noxious gas, and one as yet not much studied.* This

* See Leblanc's Researches, Comptes Rendus, vol. xxx. P. 483, and H. Bence Jones' Lectures in Med. Times and Gazette, No. 224.—Ed. Med. Ex.

bright arterial hue remains unchanged for two or three days. "This is worthy of attention; for blood is blackened by carbonic acid, yet it becomes more red upon exposure to the air, and arterial blood under the same circumstances darkens." We find it further stated, that in cases of poisoning by charcoal: "It is never the carbonic acid that kills; it is the carbonic oxide, of which but one per cent. in the atmosphere is fatal."

M. Bernard repeatedly insists on the influence of the nervous system, as necessary in the capillaries to produce the change from arterial to venous blood; or, in other words, to maintain the persistence of the chemical actions in the tissues supplied by the capillaries.

This is shown by the experiment in which the nervous communication is destroyed in a limb, and in which the blood returns in the vein of a light arterial hue; the venous color is produced again when the distal extremity of the cut nerve is stimulated by galvanism.

On the subject of temperature, there is much that will prove new and interesting to many readers.

Thus, the temperature of the blood is shown to be higher in the right ventricle than in the left; never however more than one half to one third of a degree centigrade. This departure from former opinions is explained by the fact that previous observations have been made on animals just killed, and that the greater thickness of the walls of the left ventricle does not allow the blood to cool so rapidly as in the right. M. Bernard introduced the bulb of the thermometers into the chambers of the heart of the living animal. The highest temperature is to be observed in the blood of the portal vein.

On the subject of the blood corpuscles, there is not much addition to our information; some further demonstrations are required to enable us to accept without qualification the assertion that "globulins originate the blood corpuscles."

A valuable principle in therapeutics is indicated in the remarks on albumen, the presence of which in the blood is brought forward to illustrate the manner in which any one of its constituents may act as a solvent of substances that have found their way into the circulation; they are interesting when taken in connexion with the well known observation of M. Bernard, (not herein mentioned,) of the power exhibited by the iodide of potassium to displace arsenic from the liver, and to cause it to appear in the urine during its administration.

The subject of animal sugar and its production in the economy is much more fully treated of elsewhere.

It is impossible to avoid being struck with the abundance and variety of functions assigned to the Liver, in the views of this great physiologist.

In adition to its old fashioned property of secreting bile, it it is the laboratory for the production of glucose: here albuminose is reconverted into albumen, the globules of the blood passing from it are always augmented in quantity and somewhat in character; they must have "taken their birth" in the liver, in the words of the text. Some iron disappears and the fats are destroyed in traversing this organ—while the Lungs are excused from the important part before assigned to them, for we find "The part then that the lungs perform in hæmatosis is not so great as was supposed, and we must go back to Galen's idea that the liver was the great fabricator of the blood."

In the Appendix, the observations of Robin are added concerning the blood-corpuscles, in which the description differs somewhat from that given in the earlier portion of the work. There may be some question as to the value of thus associating dissimilar accounts of different lectures; but it is decidedly objectionable to meet with contradictory accounts in the report of the same teacher as occurs in more than one instance; thus M. Bernard is made to say:

"The iron has been localised, and has been shown most positively to exist *only* in the globules." And on the opposite page:

"Iron is never found anywhere, but in the globules and in the hair;" and again: "The iron is found in less quantity in the liver than any where else."

Note C on the tissues and *parenchymes* cannot be said to give us the "last word" on that portion of Histology.

It is not our intention to enter into a prolonged criticism, but it is a source of gratification that we can refer to the recent work of Mr. Gray for a more comprehensive and accurate view of at least one parenchyma.

The spleen is stated to owe its contractibility to the presence of the muscles of organic life, and at page 198, we read, "In the walls of the spleen are many organic muscular fibres."

In man, muscular fibres have not been demonstrated in this organ. Kolliker considers the spindle shaped fibres met with in the trabeculæ not to be muscular; a view that is supported by the American translator in a note from Mr. Wharton Jones. The spleen of man would appear to derive its elasticity from the yellow fibres that enter into the external capsule and trabeculæ. According to Gray, also, no trace of muscular fibre can be detected, and he alludes to the recent experiment of Kolliker, on the spleen of a decapitated criminal, when the galvanic current failed to afford the least evidence of contraction.

Mr. Robin offers an explanation of the aggregation of the blood discs, to be observed under the microscope; and which he considers to be the exudation of a viscous fluid from their surfaces, causing them to cohere.

It is not added, why this phenomena is more generally and extensively exhibited by the blood in inflammation; and in this connection, it is to be regretted that so little space and attention is devoted to the interesting and still obscure subject of Fibrin.

In a course of lectures, it is not to be expected that subjects will be presented in the concise and systematic order to be adopted in a special treatise, and the Reporter can only give us the results of his own opportunities. The terse and absolute manner in which most of the statements are herein made, deprives the observations recorded of much of their value. There is a want of association of the subjects, an absence of generalization and inference; and but few explanations are offered, which, coming from such high authorities, would have made us realize the full merit of the investigations, besides fully impressing them on the memory.

The American author has but literally, perhaps, too literally, noted down what was said and seen, and we are brought almost into the presence of the French Professor, when we are told that he "treated *extractive matters with a shrug of the shoulders.*"

The reader cannot fail to be struck with the French idiomatic style of the work. It is very difficult, it is true, to discard the peculiar modes of expression that are acquired by frequently listening to lectures in a foreign language; but a little care in translation and revision is necessary to avoid the charge of inelegant phraseology. It requires some familiarity with the original language to appreciate the meaning of such sentences, as "life is slower," "life is quicker," "the elasticity of arteries is all," "the carbonic oxide goes away," or to supply the words wanting in this sentence, "the blood must be examined before and after each organ," in which the words, passing through, must be added to make the test intelligible.

The task of a Reporter is sufficiently difficult of itself, but when that of a Translator is added, the difficulty is certainly not diminished. It is well to bear in mind the Italian proverb, "Traduttori traditori," but in a scientific work there can certainly nothing be gained by the literal translation and introduction of such sentences as the following:

"Some who call the kidneys a gland, laugh at others who call the lungs one, but no one can laugh at the other." This is certainly not the language of serious science.

An important omission is the absence of a table of contents or index; and this is particularly felt from the desultory and disconnected order in which the subjects are treated, and recurred to more than once subsequently.

The latter portion of the work is more pleasing in its style, and has less idiomatic expressions; and although the unaccustomed words "splenic mud" startle us, as much by their inelegance as their *foreign* air, we hope that we have shown our penetration when we have translated them as "splenic pulp."

We feel much indebted for the note E, which is an abstract of a paper by M. Bernard, on the great sympathetic and the influence of its section on animal heat. It anticipates its appearance in the Comptes Rendus, and is extremely interesting as a concise summary of the experiments on that subject, and forms an agreeable conclusion to the work.

---

*A Dictionary of Medical Terminology, Dental Surgery, and the Collateral Sciences.* By CHAPIN A. HARRIS, M.D., D.D.S., Professor of the Principles of Dental Surgery in the Baltimore College, &c. *Second Edition. Carefully revised and enlarged.* Philadelphia: Lindsay & Blakiston. 1855.

Of all the weary tasks which the growing attention to literature and science has made necessary, that of the lexicographer is the most fatiguing. It is his duty to ascertain the wants of every reader, and to be at his side to supply them. He must anticipate the necessities of each individual, must go over everybody's reading, classify the whole heterogeneous mass, and smooth away the difficulties which impede the student's progress. If all this be done properly he cannot but feel the full force of Johnson's definition of a lexicographer : "A *harmless drudge*, that busies himself in tracing the original and detailing the signification of words."

There are two plans upon which a lexicon may be constructed. One of these is the plan of the cyclopædia. Each word may form the basis of an essay of greater or less extent. It will thus be composed of a series of monographs, each of which is a regular treatise, more or less elaborate, upon some special subject. Now, however desirable such a work may be, it is by no means adapted to the necessities of the student. He does not desire to interrupt his reading, at every word he does not distinctly understand, to read a dissertation upon that word. This would put upon him an undue amount of labor, distract his attention by too great a multiplicity of objects, and bewilder him in a maze of varying and contradictory theories. What he wants is a succinct and clear definition of each phrase, that he may acquire in the shortest possible time a distinct idea of its meaning. To meet his demands, the dictionary must be erected upon a basis altogether different from the cyclopædia. It must consist of a series of pointed, direct definitions, as brief as possible, consistent with precision.

The Dictionary before us has been constructed upon the latter plan. Conciseness has been attained without any sacrifice of clearness. The various words and phrases are directly defined and then dismissed. In some instances an article has been written upon a particular word, but it is always one which is of such importance as to demand this mode of treatment. There are always certain terms which comprehend much that the student often wishes a brief account of in all their relations, and finds it convenient to seek this information in his dictionary. This is the only deviation from the plan we have pointed out, and, in our opinion, the advantages derived from it justify the irregularity.

A comparison of this edition with that which preceded it, will show how great an improvement has been made. Eight thousand new words have been added, yet the size is not very greatly increased. This depends partly upon the arrangement of the types, by which a great deal of space has been economized, but chiefly upon the rejection of a large amount of comparatively uninteresting matter contained in the first edition. The author has acted very wisely in pitching overboard the bibliography and biography of dentistry. By so doing, he has made room for a large quantity of much more important matter, and has greatly increased the value of his book.

Of the manner in which the dental department has been attended to, it would be superfluous for us to speak. The high eminence of the author in that speciality, his untiring labors to advance it, and his indefatigable industry are so well known that his name is a sufficient guarantee of unsurpassable excellence.

The collateral sciences have received no little attention. In chemistry this dictionary supplies a desideratum which has long been felt. It is remarkable how this most important science has been neglected by the compilers of our standard medical lexicons. It will hardly be credited, but it is nevertheless true, that none of them which we possess, except this, contains any definition of the terms *quantitative* and *qualitative*, terms in such universal use that it would be difficult to take up a journal which does not contain them. This sort of neglect will not answer any longer, chemistry has become too intimately identified with medical science to be treated in this cavalier style. Its peculiar expressions are continually occurring in purely medical works, and the student demands their explanation. In this important department the work before us is fuller than any medical dictionary we have yet examined. It contains, as far as we can ascertain, all the important terms of the science used in medicine.

In Medical Botany, Vegetable Physiology and Zoology it is very full. Anatomy, also, both human and comparative, has been carefully considered, while the other departments of medical science have met with all the attention which could have been expected in an exclusively medical dictionary.

In short, it is a very comprehensive and satisfactory compilation, and we cordially recommend it to our readers as a safe, reliable lexicon, well adapted to the wants and requirements of the student and general medical reader.

